# Defect Detection of Aluminium Plates Based on Near-Field Enhancement of Lamb Waves Generated Using an Electromagnetic Acoustic Tranducer

**DOI:** 10.3390/s19163529

**Published:** 2019-08-12

**Authors:** Peng Zhou, Chu Zhang, Ke Xu, Weiping Ren

**Affiliations:** 1 Institute of engineering technology, University of Science and Technology Beijing, Beijing 100083, China; 2 Collaborative Innovation Center of Steel Technology, University of Science and Technology Beijing, Beijing 100083, China

**Keywords:** electromagnetic acoustic transducers, Lamb wave, near-field enhancement, aluminium plate, defect detection

## Abstract

Ultrasonic testing is an important means to detect defacing defects, such as scratches and cracks, but when the size of these defects is smaller than the wavelength of ultrasonic waves, it is difficult to detect them using traditional methods like the pulse-echo method and broadband ultrasound attenuation method for the diffraction of ultrasonic waves at the defects. Based on the non-contact characteristic of electromagnetic acoustic transducers (EMATs), a transducer for scanning inspection was developed in this paper. The transducer was utilized to detect and measure the depth of the defacing defects on an aluminium plate based on the near-field enhancement of ultrasonic Lamb waves. The results show that the amplitude of the S0 Lamb wave experiences a large enhancement when the transducer is passed over the scratch defects and the enhancement has a clearly positive correlation with the depth of the scratch defects. When the depth increases from 0.1 mm to 0.9 mm, the amplitude of S0 Lamb waves increases from 1.13 times to 2.27 times the S0 Lamb waves received on the aluminium plate without defects. The new method can be utilized to detect the defacing defects on the aluminium plate and get better detection effects than the traditional methods without analyzing the relatively small reflection waves.

## 1. Introduction

The presence of surface-breaking cracks and scratches in a mechanical structure is known to decrease its carrying capacity and resistance to corrosion, and cause stress concentration at the end of cracks that can rapidly lead to full structural failure. In practice, defacing defects usually occur at discontinuous regions of geometry or load on the component, such as a weld seam, heat affected zone, etc. Ultrasonic testing is an important means to detect these defects and measure the depth of them.

Electromagnetic acoustic transducers (EMATs) generate and detect ultrasound on electrically conducting media in a non-contact means and remove the need for a couplant, which makes them suitable for non-destructive testing (NDT) applications where the sample is at high temperatures or moving [1,2,3,4,5,6]. EMATs are electromagnetically coupled ultrasonic transducers that have complicated coupling mechanisms for ferromagnetic materials, where magnetoelastic, magnetization force, and Lorentz mechanisms contribute to transduction. In operating on non-ferromagnetic metallic materials, such as aluminium, an EMAT has only Lorentz force mechanisms to consider. The EMAT consists of a magnet, lacquered coils, and a specimen. A bulk magnet or electromagnet is employed to provide a bias magnetic field. A narrowband tone burst or wideband pulse current is commonly used to excite the coil. Meander, spiral, or racetrack coils are generally used to generate different types of ultrasonic waves. The specimen is usually restricted to a good conductor or some magnetic material. Of course, the magnet could be removed when acting as a generator, as in this article.

The pulse-echo method commonly used in ultrasonic testing employs a generator and a detector. When there are cracks on the sample, the detector receives reflection waves from cracks. The reflection waves can be used to localize and characterize these cracks via analyzing the time flight and its amplitude [7,8,9]. However, if the size of the cracks is too small to lead to an obvious reflection, it is difficult to detect those cracks using a pulse-echo method.

A laser pulse is also often used to generate ultrasonic waves in a sample, called laser ultrasound. Some researchers found that the amplitude of a surface wave experiences a large increase when either a laser source or a laser detector is passed over the defect, which is called near-field enhancement [10,11,12,13,14,15,16,17]. Using the phenomena to detect defacing defects can avoid analyzing the weak reflection waves from the defect and achieve a higher sensitivity to small-sized defects to a certain extent.

Compared to the laser ultrasound, the cost of an EMAT is cheap and the device used in the experiment is simple, so the EMAT is more suitable for the application in the industry site. However, there are still some shortcomings in the research on the near-field effect of ultrasound generated using an EMAT when the exciting coil is passed over the defect [18,19,20]. In order to simplify the problem, this paper takes the aluminium plate as a sample in which the mechanism of EMAT only includes a Lorentz force, and designs an electromagnetic acoustic transducer. The transducer is used to generate Lamb waves in the aluminium plate and perform simple scanning inspections while the other one is used to detect ultrasound waves. Compared with laser ultrasound, electromagnetic ultrasound is simpler and more suitable for applications in the industrial field.

## 2. Theory

### 2.1. Mechanism of Lorentz Force

**Figure 1:**
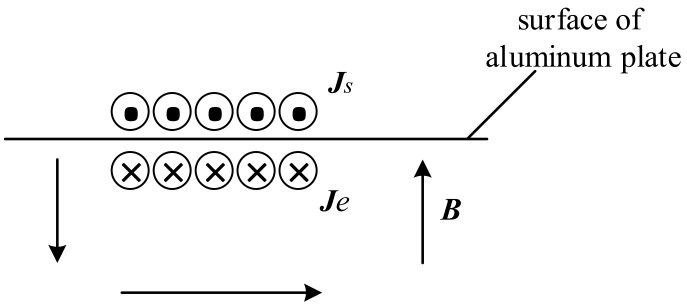
Mechanism of an EMAT in the aluminium plate.

The mechanism of EMAT non-ferromagnetic materials like the aluminium plate is the Lorentz force, which is shown in Figure 1.

When a high-frequency current, ***J**_s_*, is put into the exciting coils, a dynamic magnetic field is built around the coil according to Maxwell’s theory of electromagnetism. The magnetic field intensity, ***H***, can be solved using Equation (1) and converted to a magnetic flux intensity, ***B***, as in Equation (2), where the parameter, *μ*, is the permeability of the aluminium plate.

(1) $$\nabla \times H=J_{s}$$

(2) B=μH

While the aluminium plate is in such a dynamic magnetic field, a time-varying electric field is induced inside the aluminium plate. The electric field intensity, ***E***, can be solved using Equation (3) and converted to an eddy current, ***J**_e_*, as in Equation (4), where the parameter, *σ*, is the conductivity of the aluminium plate.

(3) $$\nabla \times E=-\frac{\partial B}{\partial t}$$

(4) $$J_{e}=\sigma E$$

The eddy current is caused by the directional movement of electrons that are subjected to the Lorentz force, ***f***, as in Equation (5). Due to the Lorentz force, particles in the aluminium plate experience a high-frequency vibration.

(5) $$f=J_{e}\times B$$

Receiving ultrasound is an inverse process of generating ultrasound in the aluminium plate. When the ultrasound propagates to the detector, the particles with the velocity ***v*** generate a time-varying current, ***J**_L_*, in the aluminium plate due to the external bias magnetic field, ***B***, as shown in Equation (6).

(6) $$J_{L}=\sigma v\times B_{0}$$

Similarly, the current, ***J**_L_*, builds a time-varying magnetic field around the receiving coil, which can be inverted to an electromotive force and a current in the coil. It can be found that the static magnetic field provided by a permanent magnet is not necessary for generating ultrasound, but is necessary for receiving ultrasound.

### 2.2. Dispersion of Lamb Waves

**Figure 2:**
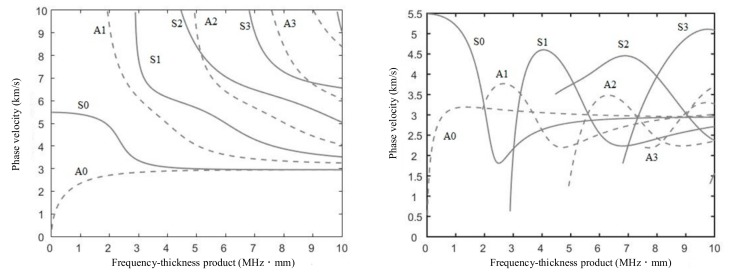
Phase velocity and group velocity dispersion curves for Lamb waves.

Lamb waves are formed by the coupling of transverse waves and longitudinal waves. According to particle vibrations in the aluminium plate, the Lamb waves can be divided into two groups: symmetric Lamb waves (s0, s1, s2, s3, …) and anti-symmetric Lamb waves (a0, a1, a2, a3, …). S0 and A0 Lamb waves are always generated regardless of the frequency, while higher order Lamb waves are only generated if the frequency–thickness product is greater than a certain threshold. For a certain mode of Lamb waves, its phase velocity and group velocity vary with the frequency in a sample with a certain thickness, which often has a bad effect on crack detection. Therefore, it is especially important to choose the appropriate current source to generate ultrasound in the aluminium plate [21,22]. The phase velocity, *C_p_*, and group velocity, *C_g_*, is a function of the frequency–thickness product, *fd*, of the system, as shown in Figure 2.

**Figure 3:**
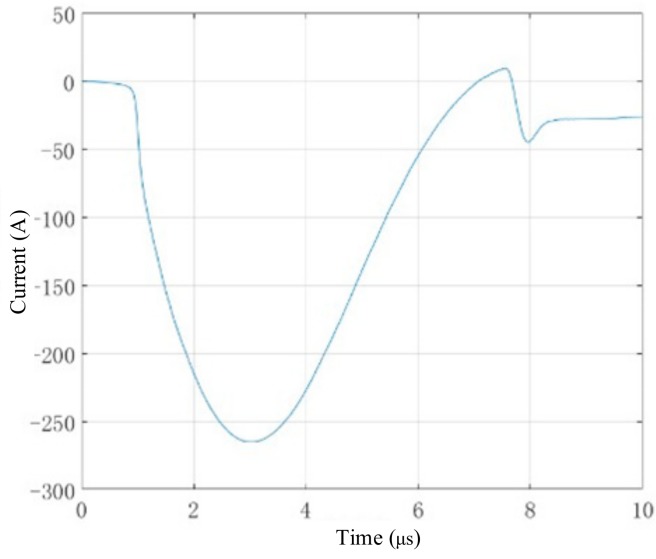
The current source through exciting coils in the experiments.

The current source used in experiments was a low-frequency and wide-band pulse generated by HPP2000 (University of Warwick, UK), as shown in Figure 3. The main frequency component was about between 0–500 kHz and the peak-to-peak value was about 270 A. On the one hand, the low-frequency pulse could only generate S0 and A0, which could avoid the interference of the higher order Lamb waves. On the other hand, the dispersion of S0 Lamb waves could generally be ignored, which is very convenient when processing the results.

## 3. Defects Inspection

### 3.1. Simulation

**Figure 4:**
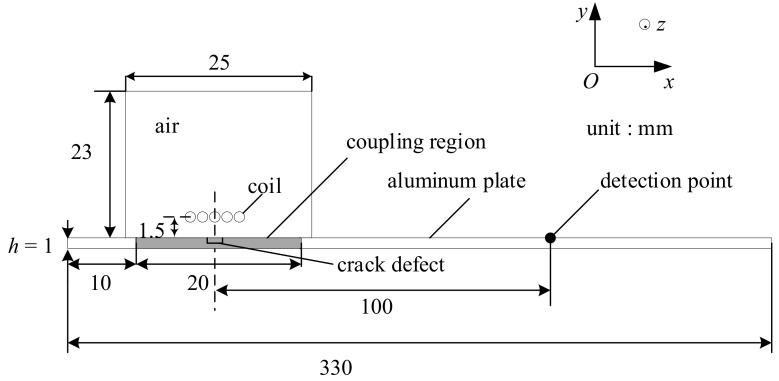
The geometry of the 2-D FE model used to simulate the near-field enhancement.

A 2-D finite element (FE) model was established to simulate the near-field enhancement of Lamb waves generated using an EMAT in the finite element software COMSOL Multiphysics 5.3 (COMSOL Inc., Stockholm, Sweden) as shown in Figure 4. In this model, the exciting coils are Φ1 mm copper wires with a 1.5 mm lift-off distance from the aluminium plate. A slot is set as the defacing defect, such as scratch and crack. The material properties of the defect area were set to aluminium or air corresponding to the aluminium plate with or without defects. The time-varying current in the exciting coil generated an eddy current $J_{e}$ and magnetic field B. Both the eddy current and magnetic field was distributed near the surface of the sample due to the skin effect. According the Equation (5), a Lorenz force was produced in the region. Therefore, the magnetic fields module and solid mechanics module in the COMSOL were applied in the simulation, and the coupling of the magnetic field to mechanics was set to a Lorenz mechanism. To cut the time consumption, the coupling region was applied in a local area right below the exciting coil rather than the whole body of the sample. A pulse of current was applied to excite the coil, as shown in Figure 4. The current signal was featured with a peak of approximately 270 A and a time range of 0–10 μs.

In order to evaluate the ultrasound waves received, this paper selected a detection point that was 100 mm far away from the center of the defect and 0.001 mm away from the top surface of the aluminium plate. In theory, the receiving coils were also required to be modeled in the simulation. However, it increased the solution time and brought an extra calculation burden. Therefore, this paper just studied the velocity at the detection point. It can be known from Equation (6) that the time-varying current ***J**_L_* in the aluminium plate was proportional to the velocity of the particle. Although the relationship between the current induced in the receiving coils and the time-varying current ***J**_L_* in the aluminium plate was not simply linear, the time-varying current ***J**_L_* could be used for qualitative analysis.

The parameter of materials used in the 2-D FE model as shown in Table 1. The air conductivity was set to a small value of 10 S/m in order to improve the stability of the solution results, which did not have a great impact on the simulation. Because only the aluminate plate regions needed to be solved when solving for the force field and the displacement field, the density, Young’s modulus, and Poisson’s ratio of air and coils did not need to be set in the model, which also reduced the time cost in calculating.

**Figure 5:**
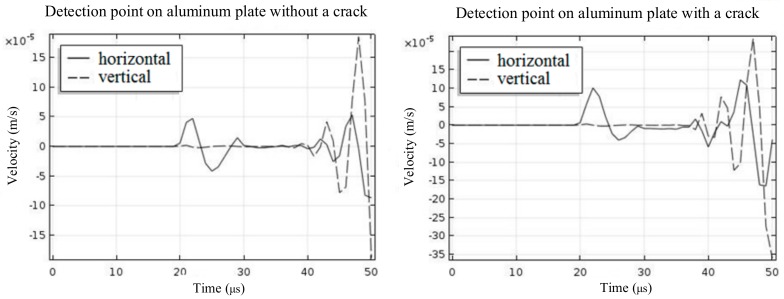
The velocity of detection point on the aluminium plate with and without cracking.

The simulation results are shown in Figure 5. Both the horizontal velocity and the vertical velocity were recorded at time intervals of 1 μs. When there was a crack on the aluminium plate, the peak-to-peak value of the horizontal velocity at the detection point clearly increased, while the vertical velocity did not change significantly. It can be inferred that the time-varying current ***J**_L_* generated by the vibration of the particle at the detection point also experienced an obvious increase. Therefore, the S0 Lamb waves received by the EMAT increased in amplitude and peak-to-peak value.

### 3.2. Scanning Inspection

**Figure 6:**
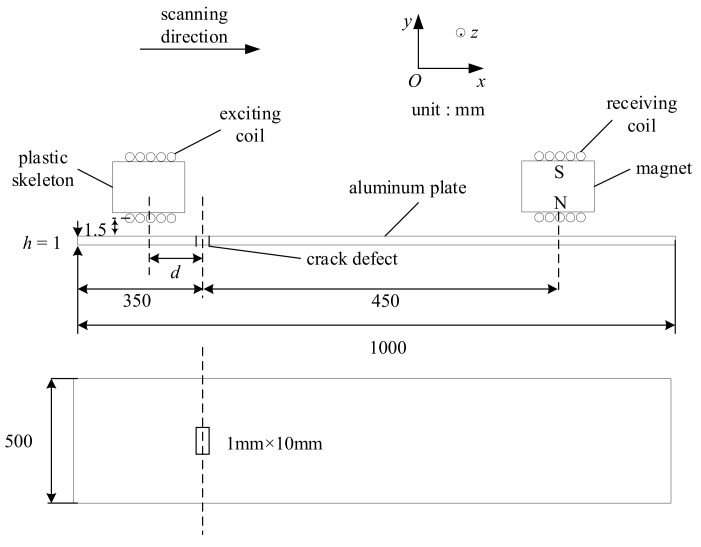
The geometry of sample and system in scanning inspection.

The experiment used a generator made up of a current source and exciting coils, and a detector made up of a permanent magnet and receiving coils. The exciting coils with a diameter of 0.68 mm were twined around a 3-D printing model with a height of 20 mm. It can be found that exciting coils were slightly different from the model established in simulation. However, the upper layer of exciting coils was 21.5 mm far away from the top surface of the aluminium plate, and therefore, the influence on the results can be ignored. Therefore, it can be considered that the EMAT was consistent with the model built in the simulation [23]. The receiving coils with a diameter of 0.1 mm were wound on a permanent magnet. During the process of a scanning inspection, the parameter, *d*, stood for the horizontal distance between the exciting coils and defect, which was from −50 mm to 50 mm with a step length of 10 mm. Furthermore, the receiving coils and permanent magnet were kept fixed, as shown in Figure 6. An artificial slot was made on the surface of the sample, which acted as the defacing defect. The slot had a size of 1 mm in width, 10 mm in length, and 1 mm in depth.

**Figure 7:**
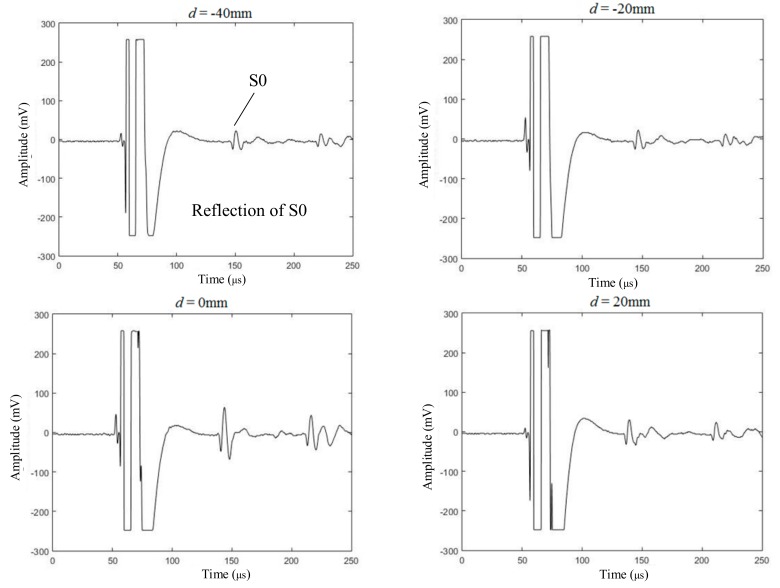

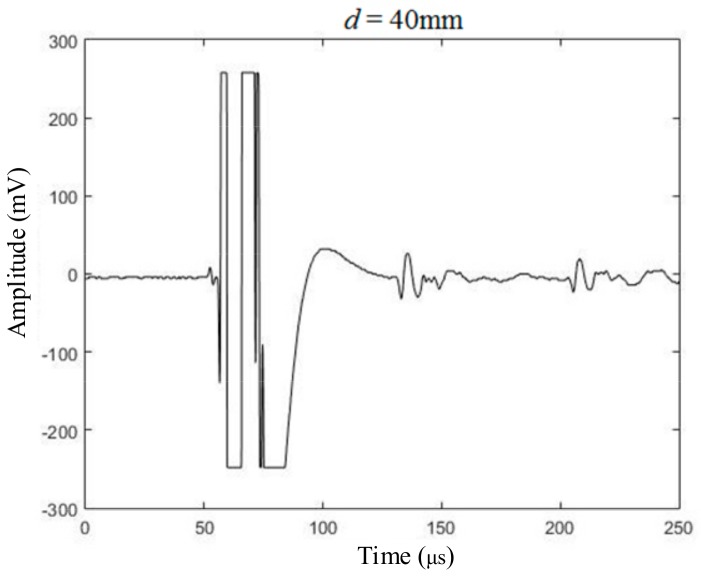
The ultrasonic waves received by EMAT in scanning inspection.

The experimental setup included a pulse generator, an EMAT generator, an EMAT receiver, a signal amplifier, a low-pass filter, and an oscilloscope. The pulse generator produced a wide-band pulse current, whose working principle was: charging an internal high-frequency capacitor first and then release the charge in a very short time to form a pulse current. An ultra-low noise signal amplifier was used to amplify the received signal with a gain of about 80 dB. A low pass filter was applied to filter out high-frequency noise signals (higher than 1 MHz). An oscilloscope (Tektronix, TBS 1102B, Beaverton, Oregon, OR, USA) acted as the signal collector, which could be replaced by a dedicated signal acquisition and analysis system for automatic measurement. Some of experimental results are shown in Figure 7. When *d* equals 0 mm, which means that the exciting coils scan above the crack, the peak-to-peak value of S0 Lamb waves received by the receiving coils experienced an obvious enhancement. The experimental results were consistent with the simulation to a certain degree.

In order to quantitatively analyze the experimental results, the relationship between the parameter, *d*, and the peak-to-peak value of the S0 Lamb waves is shown in Table 2. When *d* was less than zero, which meant the exciting coils and the receiving coils were on the different sides of the defect, the S0 Lamb waves experienced an attenuation when it encountered the defect in the propagation. Furthermore, when *d* was greater than zero, which meant the exciting coils and the receiving coils were on the same side of crack, the reflected and forward S0 Lamb waves were overlapped. Therefore, it can be found in Table 2 that the peak-to-peak value of the S0 Lamb waves given that *d* was less than zero was slightly less than the peak-to-peak value of the S0 Lamb waves given that *d* was greater than zero. Due to the diffraction of the ultrasonic wave, it was difficult to detect the small-size crack using the conventional pulse-echo method. Based on the near-field enhancement of the S0 Lamb waves generated by the EMAT, the scanning inspection method could be used to detect and position the crack using the position of the exciting coils.

## 4. Depth Measurement

Ultrasonic testing is an important means for the quantitative measurement of the depth of defacing defects, such as the tip diffraction method, creeping wave testing, time of flight diffraction, etc. These methods usually use the diffraction of ultrasonic waves at the tip of the defect to evaluate the depth. However, when the depth of the defect is small, it is difficult to separate the diffracted waves from the forward ultrasonic waves. The near-field enhancement of the S0 Lamb waves was discussed in Section 3, and the depth of the defect had a great influence on the enhancement of the ultrasonic amplitude. The increasing coefficient of the amplitude is called the near-field enhancement coefficient.

### 4.1. Simulation

**Figure 8:**
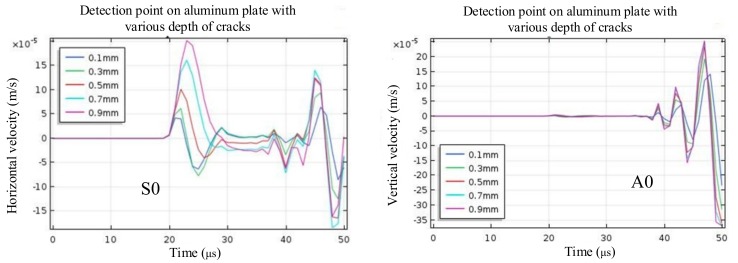
The velocity of the detection point on aluminium with various crack depths.

In order to study the influence of depth on the near-field enhancement coefficient, A 2-D FE model was established in the finite element software COMSOL Multiphysics 5.3, as shown in Figure 4. The depth of the defect was set to 0.1 mm, 0.3 mm, 0.5 mm, 0.7 mm, and 0.9 mm, and other simulation parameters remained the same. When the depth of the defect increased, the horizontal velocity of the detection point also increased gradually, and the vertical velocity A0 waves did not change significantly, as shown in Figure 8. Therefore, the amplitude of S0 Lamb waves also increased with the increase of depth, and the near-field enhancement of the S0 Lamb waves became greater.

### 4.2. Experiments at Different Crack Depth

**Figure 9:**
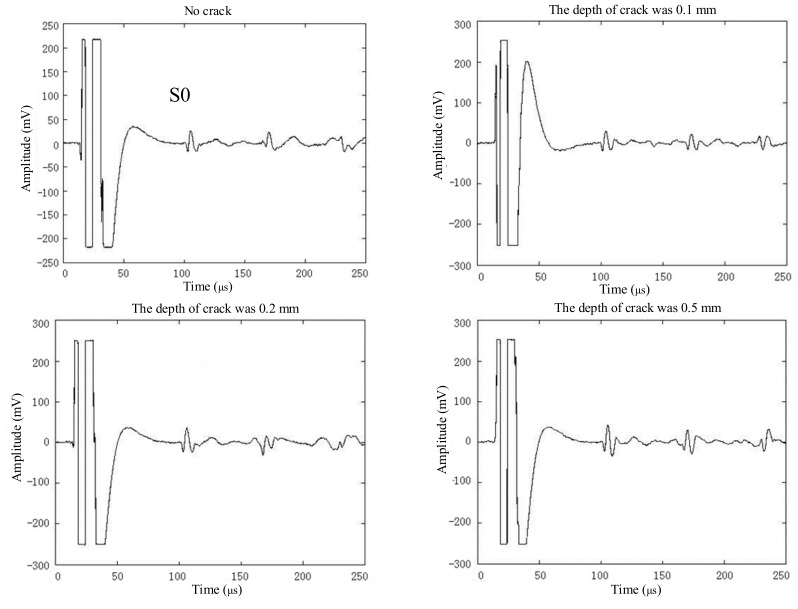

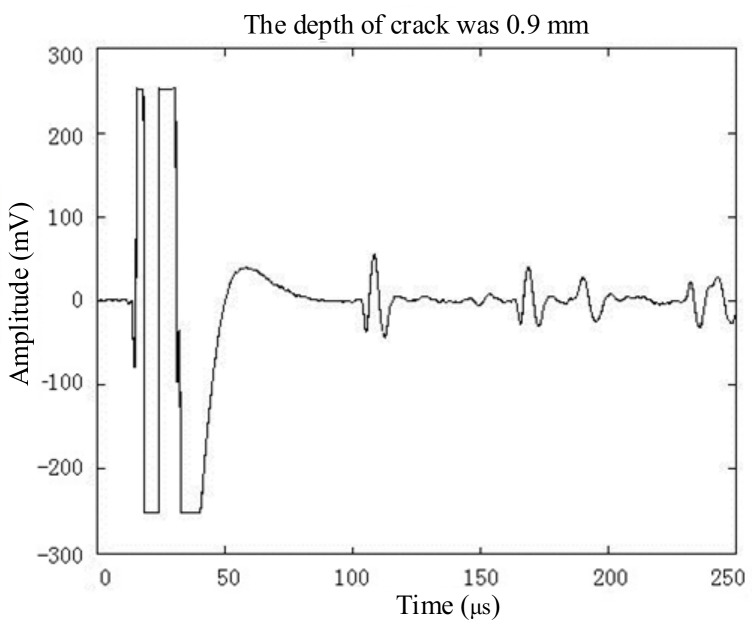
The ultrasonic waves received by the EMAT during the scanning inspection.

In this paper, slots with a depth of 0.1 mm, 0.2 mm, 0.5 mm, and 0.9 mm were artificially fabricated on the aluminium plate. The exciting coils were placed directly above the defect, and the receiving coils were 450 mm away from the exciting coils. The experimental results are shown in Figure 9. It can be found that the near-field enhancement of the S0 Lamb waves was positively correlated with the depth of the defect.

**Figure 10:**
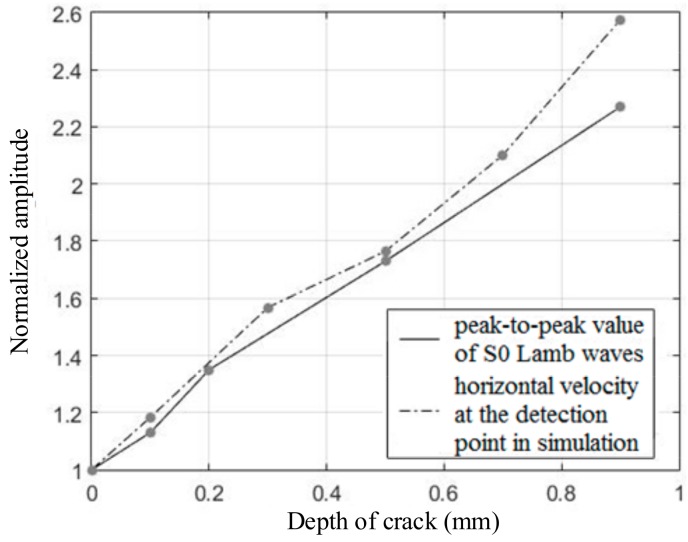
The influence of depth on the near-field enhancement coefficient of S0 Lamb waves.

In order to quantitatively analyze the experimental results, the relationship between the depth of the defect and the normalized peak-to-peak value of the S0 Lamb waves was studied and is shown in Figure 10. As the depth of the defect increased, the enhancement coefficient of the S0 Lamb waves also increased, and the peak-to-peak value of the S0 Lamb waves gradually increased too. When the depth increased from 0.1 mm to 0.9 mm, the enhancement coefficient increased from 1.13 to 2.27. Although there were a few differences between the experimental results and the horizontal velocity at the detection point in the simulation, the overall variation trends remained the same. The results showed that the enhancement coefficient could be used to measure the depth of the crack or evaluate the severity of the crack after calibration in advance.

## 5. Discussion

There are three main reasons leading to the near-field enhancement of S0 Lamb waves. First of all, the material loss changes the distribution of the electromagnetic field and eddy current inside the aluminium plate in the coupling region, as shown in Figure 4, which may increase the magnetic flux intensity, ***B***, and the eddy current, ***J****_e_*, at the tip of the defect. Second, some of the ultrasonic waves are reflected when encountering the defacing defect during propagation, which lead to the overlap of forward waves and reflected waves. The distance between the exciting coil and the defect is so close that they arrive at the detector at almost the same time. Finally, when there is a defacing defect on the aluminium plate, the boundary conditions at the edge of the defect become condition free from the continuous conditions. The vibration in the coupling region increases given the same Lorentz force and the ultrasound propagating in the aluminium plate also experiences an enhancement.

The current source used in experiments was a low-frequency and wide-band pulse, whose main frequency component was about between 0–500 kHz, as shown in Figure 3. The group velocity of S0 Lamb waves is about 5500 m/s in 1 mm-thick aluminium plate. In can be inferred that the wavelength of S0 Lamb waves is about 11 mm greater than the size of the crack, as shown in Figure 6. Therefore, the reflection of the S0 Lamb waves at the crack was not obvious, and it was difficult to detect the defect using the pulse-echo method. The scanning inspection method was less dependent on the reflected waves compared to the pulse-echo method, which was practical in detecting a smaller defacing defect using the scanning inspection method given the same current source with a low frequency.

There were three main reasons that led to the difference between the enhancement coefficient of S0 Lamb waves and the horizontal velocity at the detection point in the simulation, as shown in Figure 10. First of all, the S0 Lamb waves received by the detector were not only related to a certain point, but a region. However, the enhancement coefficient of the horizontal velocity just took the detection point into consideration. Second, the distance between the detection point and the center of the defect in simulation was 100 mm in order to reduce the calculation cost. However, the distance in depth measurement was 450 mm in order to separate the electromagnetic interference and S0 Lamb waves in the time domain, as shown in Figure 7 and Figure 9. Therefore, the different distances may have had an influence on the enhancement coefficient. Finally, it can be known from Equation (6) that the time-varying current ***J**_L_* in the aluminium plate was proportional to the velocity of the particle. However, the relationship between the current induced in the receiving coils and the time-varying current ***J**_L_* in the aluminium plate was not simply linear; this may introduce some errors when using the enhancement coefficient of horizontal velocity to evaluate the enhancement coefficient of the S0 Lamb waves.

## 6. Conclusions

(1) There were only S0 and A0 Lamb waves given the current source with a 0–500 kHz main frequency component in a 1 mm-thick aluminium plate. Furthermore, under this condition, the dispersion of S0 Lamb waves was relatively weak and negligible, which is suitable for defect detection.
(2) The scanning inspection method based on the near-field enhancement of S0 Lamb waves could be used to detect and position the 1 mm × 10 mm defacing defect on the 1 mm-thick aluminium plate more effectively compared to traditional ultrasonic testing like the pulse-echo method.
(3) As the depth of the defacing defect increased, the near-field enhancement coefficient of the S0 Lamb waves also experienced a clear increase, whose overall trend was consistent with the simulation results. The near-field enhancement coefficient could be used to measure the depth of the defect or evaluate the severity of the defect after calibration in advance.

## Figures and Tables

**Table 1:** Parameters of samples employed in the 2-D FE model.

	Air	Coil	Aluminium
Relative permeability	1	1	1
Relative permittivity	1	1	1
Conductivity	10 S/m		3.774 × 10^7^ S/m
Density			2700 kg/m^3^
Young’s modulus			70 GPa
Poisson’s ratio			0.33

**Table 2:** The relationship between *d* and the peak-to-peak value of the S0 Lamb waves.

*d*	S0 Lamb Peak	*d*	S0 Lamb Peak
−50 mm	48 mV	10 mm	66 mV
−40 mm	48 mV	20 mm	64 mV
−30 mm	44 mV	30 mm	62 mV
−20 mm	48 mV	40 mm	58 mV
−10 mm	44 mV	50 mm	56 mV
0 mm	132 mV		
